# Price Current and Notes on New Remedies

**Published:** 1884-03

**Authors:** 


*Price Current and Notes on New Remedies.* FERRIS & CO., Bristol.

Our enterprising fellow-townsmen have sent us a large closely-printed quarto volume of 130 pages, containing a full list of all their medicines, instruments and specialities, with a full posological table and extensive notes on new drugs. The notes on recently introduced remedies, extending to nearly fifty pages, are carefully compiled from current periodicals, and present in convenient and readily accessible form all the reliable information which the practitioner needs to guide him in prescribing them.

